# Tea polyphenols inhibit the proliferation, migration, and invasion of melanoma cells through the down-regulation of TLR4

**DOI:** 10.1177/0394632017739531

**Published:** 2018-01-23

**Authors:** Xianjin Chen, Lili Chang, Yan Qu, Jinning Liang, Waishu Jin, Xiujuan Xia

**Affiliations:** 1 Department of Dermatology, The Affiliated Yantai Yuhuangding Hospital of Qingdao University, Yantai, China; 2 Department of Cardiac Surgical Care Unit, The Affiliated Yantai Yuhuangding Hospital of Qingdao University, Yantai, China

**Keywords:** melanoma, tea polyphenols, TLR4

## Abstract

Melanoma is the most common skin cancer and malignant melanoma which can cause skin cancer-related deaths. Toll-like receptor 4 (TLR4) had been reported to play an important role in melanoma, and tea polyphenol (TP) is regarded as an anticancer substance. However, the relationship between TP and TLR4 in melanoma is not well explored. Therefore, our aim is to figure out how TP has an influence on melanoma. Melanoma cell lines (B16F10 and A375) were treated with TP and lipopolysaccharides (LPS). Western blot assay was used to examine TLR4 expression, and MTT assay was conducted to assess proliferation. Wound healing assay was conducted to evaluate the migration of melanoma cells, and transwell assay was used to examine the melanoma cells’ invasiveness. Besides, in vivo experiments were practiced for TP function in mice with melanoma cells. TP inhibited the proliferation, migration and invasion ability of melanoma cells, which displayed a dosage and time dependence. TLR4 was highly expressed in melanoma cells compared with normal skin cells. TP could suppress TLR4 expression both in normal melanomas and in stimulated melanomas by TLR4 agonist LPS. Suppressing TLR4 in melanomas could inhibit cell function (proliferation, migration, and invasion), and blocking the expression of 67LR could abolish TP function on TLR4. TP can inhibit melanoma (B16F10) growth in vivo.

## Introduction

Melanoma, also known as malignant melanoma, is a type of cancer that develops from the pigment-containing cells known as melanocytes.^1^ In 2015, 3.1 million were diagnosed and 59,800 died with the active disease.^2,3^ Environmental factors (such as ultraviolet light (UV)) are recognized to be the main cause of melanoma.^4^ In addition, epigenetic alterations which alter the expression levels and functioning of tumor suppressor genes are also a major cause.^5^ Finding effective ways to decline the risk of melanoma has become a great concern of the world.

Tea contains various phenolic contents including phenols, polyphenols, and natural plant compounds. Catechin, as an important player in polyphenols, can be divided into epigallocatechin-3-gallate (EGCG), epicatechin (EC), epicatechin-3-gallate (ECg), epigallocatechin (EGC), and gallocatechin (GC). EGCG accounts for 50%–80% of the catechin.^6^ Enormous clinical studies and laboratory animals had revealed the function of tea polyphenols (TPs).^7,8^ For instance, green TP could inhibit tumor cells growth and survival;^9^ TP could suppress melanoma growth by inhibiting IL-1beta secretion.^10^ In this study, we determined to investigate the function of TP in melanoma and the specific mechanism.

Toll-like receptors (TLRs) are recognized as pattern recognition receptor proteins which help defend the invading pathogens.^11^ The study has demonstrated that TLRs are expressed in keratinocytes and melanocytes, the main part of the skin and arising-expression in skin cancers.^12^ Toll-like receptor 4 (TLR4) is a member of the TLRs family and has been widely studied for its ability to fight many diseases.^13^ In TLRs family, TLR4 is frequently studied for its ability to fight many diseases. The role of TLR4 is evaluated that TLR4 can induce dendritic cells, activate environmental danger molecules, and inhibit melanoma.^14,15^ It has been proven that melanoma is regulated by interfering TLR4 signals.^16^ In addition, some studies show that EGCG can inhibit TLR4 expression or inhibit the TLR4 signaling pathway.^17,18^ This study then aimed to discover the possible relationship between TP and TLR4 in melanoma treatment.

This study evaluated overall effects of TP on melanoma cells by investigating proliferation, migration, and invasion ability changes as TP concentration grew. It also investigated TP/TLR4 connection and their co-function on melanoma cells. Through the mechanism study, we may enlighten future melanoma therapy.

## Materials and methods

### Cell culture and drug treatment

Melanoma cell lines (B16F10 and A375) and normal skin cells (JB6 and HaCaT) were obtained from the Shanghai Cell Bank of Chinese Academy of Sciences. Cells were cultured in a Dulbecco’s modified eagle medium (DMEM) containing 10% fetal bovine serum (FBS), penicillin (100 U/mL), and streptomycin (100 μg/mL) at 37°C in a culture chamber which contained 5% CO_2_. In all experiments, cells were allowed to acclimate for 24 h before any treatment.

Melanoma cell lines (B16F10 and A375) were treated with TP (5, 10, 20, and 40 μg/mL; Solarbio, Beijing, China, # T1090) and TLR4 agonist lipopolysaccharides (LPS; 100 ng/mL; Invitrogen, San Diego, CA, USA, cat.code: tlrl-ppglps). The negative control (NC) group was treated with phosphate buffer solution (PBS) for TP or LPS, and the Mock group was treated with the transfection reagents (Lipofectamine 3000) for small interfering ribonucleic acid (siRNA) or short hairpin ribonucleic acid (shRNA).

shRNA for 67LR, 5′-GGAGGAATTTCAGGGTGAA-3′. The annealed shRNA inserts were cloned into the psiRNA-hH1neo shRNA expression vector (for 67LR-shRNA) (Invitrogen, San Diego, CA, USA) according to the manufacturer’s protocol. siRNA (10 µmol/L) for TLR4, 5′-CCTTTCCGGGACTTTCGCTTT-3′, was ordered from Thermo Fisher Scientific. Lipofectamine 3000 (Thermo Fisher Scientific, USA) Thermo Fisher Scientific (Waltham, MA, USA) was used to transfect RNAs in melanoma cells.

### MTT assay

Approximately, 4 × 10^3^ cells/well were plated in flat-bottom 96-well plates and treated with 200-μL TP (5, 10, 20, and 40 μg/mL) 24 h after plating. After 48 h of treatment, the supernatant was removed and cells were incubated for 4 h with the MTT reagent (300 μL; 5-mg/mL final concentration in medium; Abnova, Taiwan, #KA1606). The MTT was then dissolved by adding 150-μL dimethyl sulfoxide (DMSO), and absorbance was recorded at 490 nm using an enzyme-linked immunosorbent assay (ELISA) reader. The experiment was repeated three times.

### Wound healing assay

Cell suspension in the logarithmic phase was seeded in a 6-well plate (2 mL, 2 × 10^5^ cells per mL) and cultured for 24 h. A straight line was scratched using a sterile pipette tip (10 μL) on the surface of cells when cells reached 80%–90% confluence. After being washed with PBS three times, 2 mL of DMEM with 2% FBS at different concentrations of TP (5, 10, 20, and 40 μg/mL) was added. The migration situations of cells in different groups were observed at 0, 12, and 24 h after incubation. Analysis and calculation was conducted using IPP6 software. The experiments of each group were repeated three times.

### Transwell assay

Cell suspension (200 μL, 2 × 10^6^ cells per mL) was added into the upper well, and 700 μL of DMEM with 15% FBS and different concentrations of TP (5, 10, 20, and 40 μg/mL) was added into the lower well. Cells were cultured at 37°C for 24 h and then cells on the surface of the upper well were removed. The membrane was fixed with paraformaldehyde (4%) for 30 min and subsequently stained with 0.1% crystal violet for 15 min. After being washed three times with PBS, cells on the lower surface were observed through a microscope. The experiments were repeated a minimum of three times.

### Western blotting analysis

When observing the different concentrations of TP influence on TLR4, cell suspension in the logarithmic phase was seeded in a 6-well plate (2 mL, 5 × 10^5^ cells per mL), and different concentrations of TP (5, 10, 20, and 40 μg/mL) were incubated for 24 h. TP (40 μg/mL) was chose to treat cells for 0, 6, 12, and 24 h. Cells were also lysed with ice-cold lysis buffer (50-mM Tris-HCl, pH 6.8, 100-mM 2-mercaptoethanol, 2% w/v sodium dodecyl sulfate, and 10% glycerol). Proteins were separated with 10% sodium dodecyl sulfate–polyacrylamide electrophoresis and then transferred onto a polyvinylidene difluoride (PVDF) membrane at 100 V for 1.5 h. Proteins were treated with Tris-buffered saline and Tween which contained 5% non-fat dried milk for 1 h. Blots were incubated with primary antibodies (Rabbit Anti-human TLR4, 1:800, Cell Signaling) at 4°C overnight. This was followed by incubation with secondary antibodies (HRP-labeled Goat Anti-Rabbit IgG (H + L), 1:2000, Cell Signaling) at room temperature for 2 h. After being washed with PBST (phosphate buffered saline + 1% Tween 20), blots were analyzed using Gel-Doc 200 (Bio-Rad). Bio-Rad (Hercules, CA, USA) GAPDH. GAPDH: glyceraldehyde phosphate dehydrogenase was used as the internal reference.

### Animal green tea supplementation and tumor growth in vivo

Four-week-old male C57BL/6Js weighing 150 ± 40 g were purchased from the experimental animal center of Qingdao University. They were divided into two groups, namely, the tumor group and the TP injection group, and three mice were finally selected for comparison. All animal procedures and experimental protocols were approved by the Laboratory Animal Ethics Committee of The Affiliated Yantai Yuhuangding Hospital of Qingdao University. A total of 5 × 10^6^ B16F10 cells in 200 μL medium were subcutaneously injected into each mice. After 5 days, one group was randomly selected for TP gavage (500 mg/kg of body weight, solubilized in water). The other group gavage fed with water according to body weight and tumors were measured using vernier calipers every 5 days. Tumor volumes were also calculated according to the following formula: volume = (length × width^2^)/2. Gavage was performed every 2 days for 30 days. On the 30th day, all mouse were sacrificed via cervical dislocation, and the tumors were dissected, weighed, and frozen at −80°C for further work.

### Statistical analysis

Statistical analysis was achieved using the GraphPad Prism 6.0 software (Chicago, IL, USA). Experiments were repeated three times, and results were presented as mean ± standard deviation (SD). Differences between treatments were tested by student’s t test and one-way analysis of variance (ANOVA). *P* < 0.05 was considered a statistical difference.

## Results

TP suppressed melanoma cells ability with dosage dependence.

**Figure 1.**
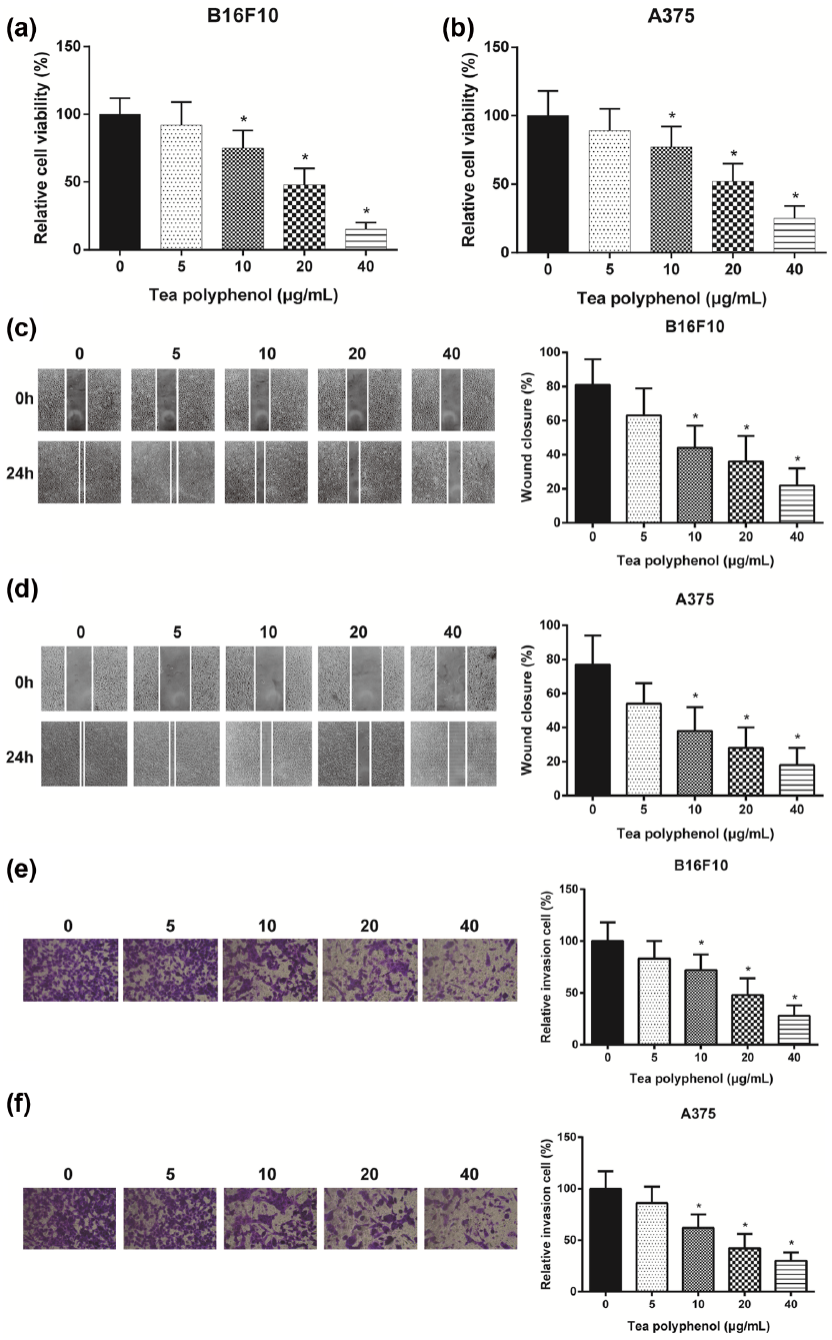
TP suppressed melanoma cells ability: (a and b) cell proliferation decreased significantly as TP concentration grew by MTT assay. Cell viability decreased significantly compared with non-TP group as TP concentration grew. (c and d) Cell migration decreased significantly as TP concentration grew by wound healing assay. Smaller wound closure was detected as TP concentration grew, indicating fewer cells migration, and (e and f) cell invasion decreased significantly as TP concentration grew by transwell assay. Less invasion cells were detected in higher concentration TP group. *Significant difference compared with non-TP group with *P* < 0.05.

B16F10 and A375 cells were treated with TP (5, 10, 20, and 40 μg/mL) for 48 h and then cell viability was tested. As demonstrated by the MTT assay, the viability of cells treated with TP (5 μg/mL) displayed no significant changes (*P* > 0.05). However, the group with higher concentration (10, 20, and 40 μg/mL) of TP presented remarkable reduction in both B16F10 cells and A375 cells (*P* < 0.05, Figure 1(a) and (b)). This result demonstrated that TP inhibited melanoma cells proliferation and the inhibition rose with concentrations. Migration rate also displayed the same concentration dependent trend considering decreasing wound closure (*P* < 0.05, Figure 1(c) and (d)). In addition, transwell assay revealed that TP could inhibit cell invasion, and the inhibition grew with increasing concentrations (*P* < 0.05, Figure 1(e) and (f)). All those results indicated that TP inhibited the proliferation, migration, and invasion of melanoma cells, and the inhibition was dose-dependent.

### TP suppressed TLR4 expression in melanoma cells

**Figure 2.**
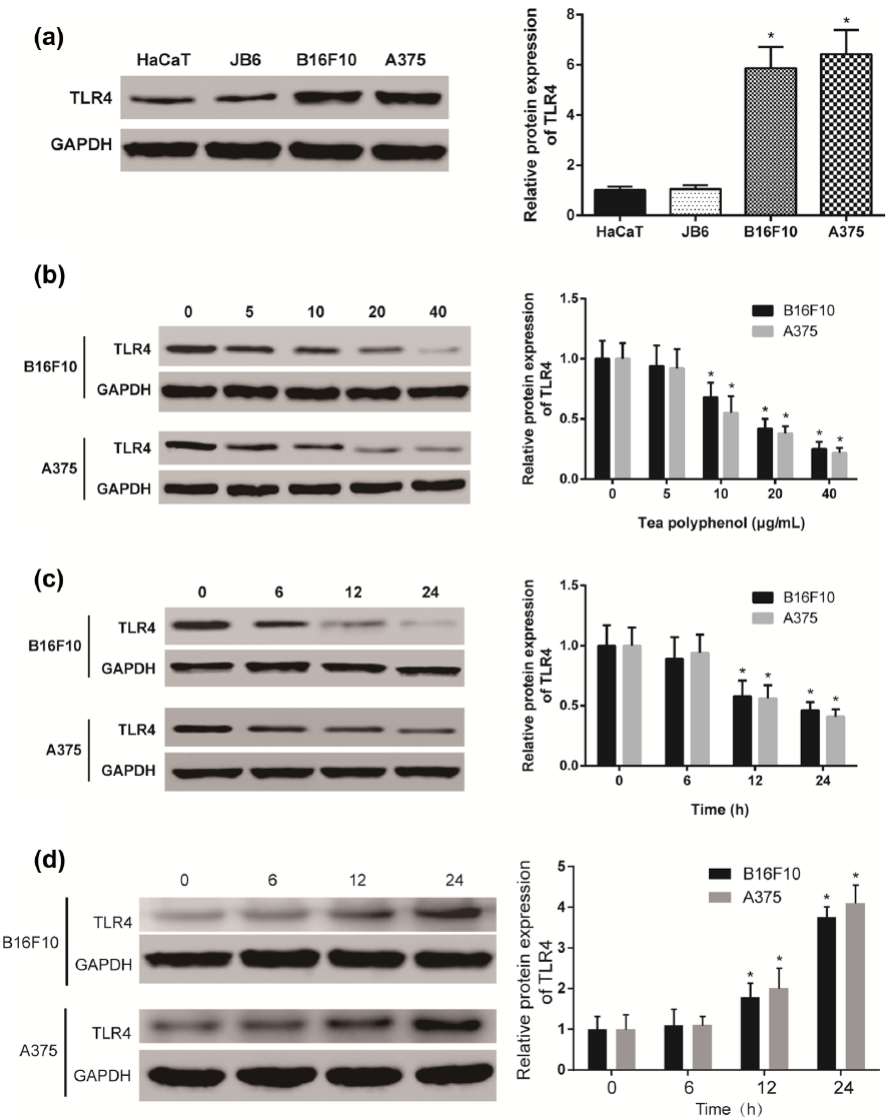
TP suppressed TLR4 expression in melanoma cells: (a) TLR4 was high expressed in melanoma cell lines B16F10 (mouse) and A375 (human) compared with normal skin cell lines HaCaT (human) and JB6 (mouse). (b) TP decreased protein expression of TLR4 significantly and displayed dosage dependence. Higher TP concentration resulted in lower TLR4 expression in B16F10 and A375 cell lines (*significant difference compared with non-TP group with *P* < 0.05). (c) TP decreased protein expression of TLR4 significantly and displayed time dependence. Longer treatment of 20 μg/ml TP led to less TLR4 expression in B16F10 and A375 cell lines (*significant difference compared with 0 h with *P* < 0.05), and (d) removal of TP increased TLR4 protein expression and displayed time dependence. Longer recovery led to higher TLR4 protein expression (*significant difference compared with 0 h with *P* < 0.05).

Western blot results showed that the protein of TLR4 expression in melanoma cells, B16F10 (mouse) and A375 (human), was significantly higher than that in normal skin cells, HaCaT (mouse) and JB6 (human) (*P* < 0.05, Figure 2(a)). After 24 h treatment, TLR4 protein expressions were detected at different TP concentrations. TLR4 expression displayed no significant changes in the TP (5 μg/mL) group (*P* > 0.05). However, TLR4 expression in higher TP concentration groups was lower (*P* < 0.05, Figure 2(b)). To further confirm the inhibition mechanism of TP on TLR4 expression, 20 μg/mL TP was used to treat melanoma cells for 6, 12, and 24 h. The results showed that TLR4 expressions in the 12- and 24-h TP treated groups significantly decreased (*P* < 0.05, Figure 2(c)). In conclusion, TP inhibited TLR4 expressions in melanoma cells (B16F10 and A375). After TP was removed, TLR4 expression recovered and displayed concentration dependence (*P* < 0.05, Figure 2(d)). From the results shown above, TP could suppress TLR4 in melanoma, and the suppression strengthened with concentration increase.

### TP acted on melanoma through TLR4 suppression

**Figure 3.**
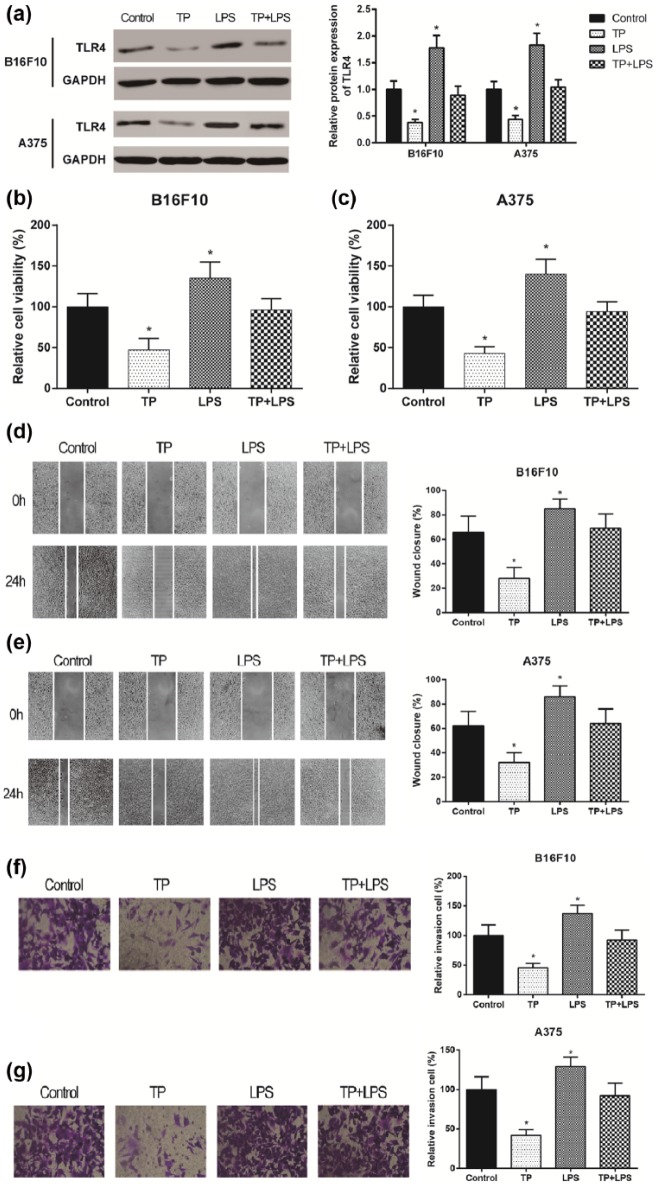
TP acted on melanoma through TLR4 suppression with LPS application: (a) TP suppressed TLR4 expression while LPS, the agonist of TLR4, improved LPR4 expression in B16F10 and A375 cell lines by western blot. (b and c) Cell viability of melanoma cells was decreased by TP while increased by LPS. (d and e) Cell migration of melanoma cells was decreased by TP while increased by LPS, and (f and g) cell migration was decreased by TP while increased by LPS. *Significant difference compared with control group with *P* < 0.05.

LPS is an agonist which up-regulated TLR4 expression significantly. Cells were divided into four groups (Control/TP/LPS/TP + LPS). Western blot showed that TP inhibited TLR4 expression but LPS stimulated TLR4 expression while no significant changes displayed in TP + LPS group (*P* > 0.05, Figure 3(a)). MTT assay results showed that cell proliferation significantly reduced in the TP group and increased in the LPS group (both *P* < 0.05). However, cell proliferation in TP + LPS group was similar to that in the control group (*P* > 0.05, Figure 3(b) and (c)). Wound healing then demonstrated decreased migration rate in TP group and increased migration rate in LPS group along with standing rate in TP + LPS group (*P* > 0.05, Figure 3(d) and (e)). Besides, transwell assay displayed decreased invading number in TP group and increased invading number in LPS group (*P* > 0.05, Figure 3(f) and (g)). Above all, TP could suppress the proliferation, migration, and invasion of melanoma through TLR4 suppression.

**Figure 4.**
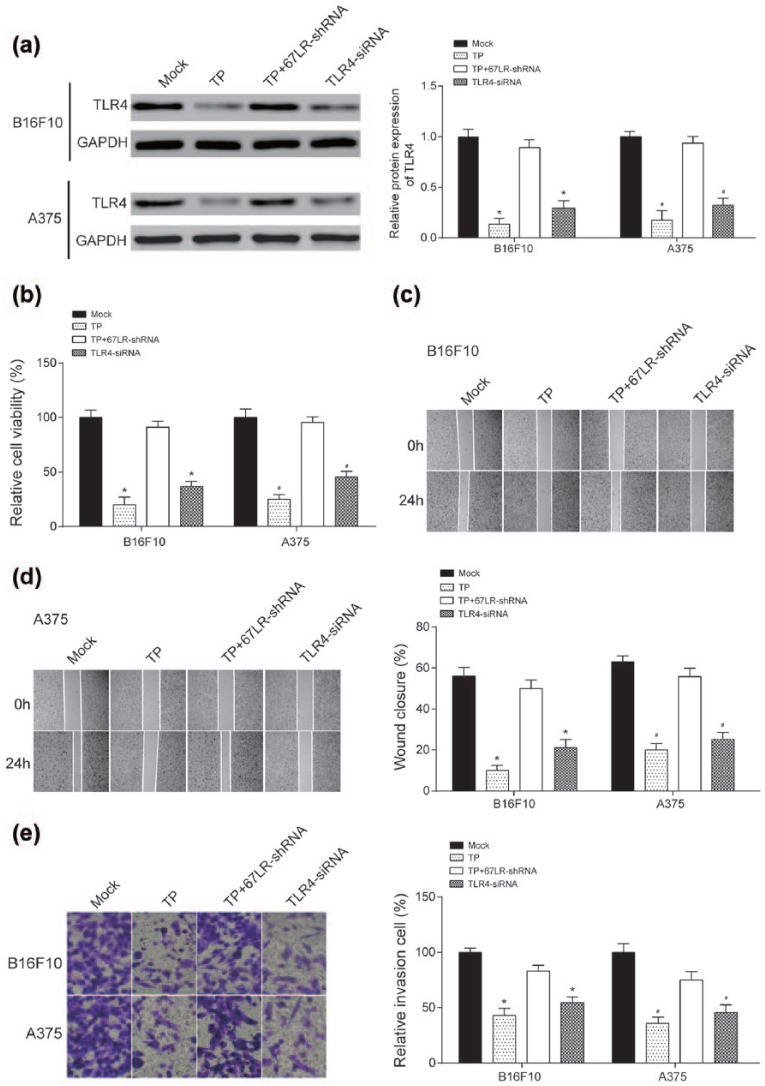
TP acted on melanoma through TLR4 suppression with 67LR-shRNA and TLR4 siRNA application: (a) TP and TLR4 siRNA suppressed TLR4 expression significantly, and TP+67LR-shRNA could reverse the process by western blot. (b) Cell viability of melanoma cells was decreased by TP and TLR4 siRNA but reversed by TP+67LR-shRNA by MTT assay. (c and d) Cell migration of melanoma cells was decreased by TP and TLR4 siRNA but reversed by TP+67LR-shRNA by wound healing assay, and (e) cell invasion was decreased by TP and TLR4 siRNA but reversed by TP+67LR-shRNA by transwell assay. *(BB16F10) and #(A375) indicate significant difference compared with control group with *P* < 0.05.

TLR4 siRNA and 67LR-shRNA were constructed, respectively, to knockdown TLR4 expression and 67LR expression, and 67LR was the receptor of TP. According to western blot, TP and TLR4 siRNA down-regulated TLPR4 expression and TP+67LR-shRNA up-regulated TLR expression (*P* < 0.05, Figure 4(a)). Furthermore, in TP/TLR4 siRNA groups, the proliferation of melanomas were remarkably weaker (*P* < 0.05, Figure 4(b)), the migration of melanomas were significantly lower (*P* < 0.05, Figure 4(c) and (d)), and the invasive melanoma cells were prominently decreased, all compared with group of Mock (*P* < 0.05, Figure 4(e)). However, TP abolished the inhibitive function on TLR4 when blocked the 67LR in melanomas, and the same phenomenons appeared in the results of MTT assay, wound healing assay, and Transwell assay (*P* > 0.05, Figure 4(b)–(e)).

### TP inhibited tumor growth in vivo

**Figure 5.**
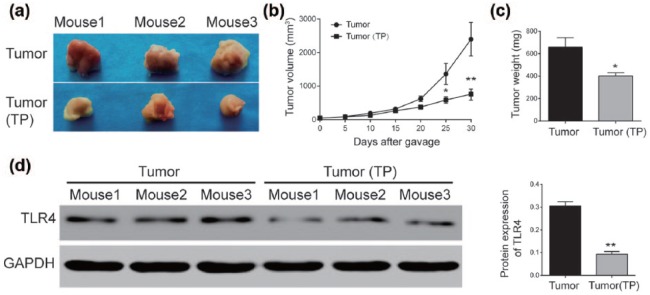
TP inhibited tumor growth in vivo: (a and b) tumor volume was significantly smaller in TP group compared with the tumor group. (c) Tumor weight on the 30th day was significantly smaller in TP group compared with the tumor group, and (d) TLR4 protein expression drastically decreased after TP injection. *Significant difference compared with control group with *P* < 0.05.

At the same time, whether TP could suppress melanoma cells (B16F10) growth, primary experiment in vivo was conducted, the results showed that the tumors sizes were smaller in TP groups than in groups with water (*P* < 0.05, Figure 5(a) and (c)). Figure 5(b) showed significant decreases in TP group in tumor volume (*P* < 0.05, Figure 5(b)), and TLR4 protein also displayed a significant drop in TP group (*P* < 0.05, Figure 5(d)).

## Discussion

Natural polyphenols exists in fruits, vegetables, cereals, and tea and they influenced the pathology of a variety of diseases.^19^ Many reports have revealed TP as a tumor inhibitor. For instance, some studies confirm that green tea extracts can delay cancer cell migration in hepatocellular carcinoma cells (HepG2).^7^ Furthermore, TP also has anti-proliferative effects in lung carcinoma (A549) and cervical carcinoma (HeLa) cells by suppressing NF-κB activation and the expression of cyclin D1.^20^ TP can improve the melanomas treatment efficacy.^21^ Related studies have shown that green TP can inhibit the growth of melanoma cells by down-regulating IL-1β secretion.^10^ Results show that TP has dose- and time-dependent effects on melanomas, and those effects are reported in many substances.^22^ Like honey and chrysin, it can reduce the proliferation of melanoma cells.^23^ TLR4 signaling was proven to promote melanoma progression.^24^

This research showed that TP could inhibit melanoma cells function through TLR4 suppression, which displayed dose and time dependence. TP plays a pivotal role in TLR4 suppression by suppressing the activation of the TLR4 signal pathway.^6,8^ To explore the possible underlying mechanism, Hong et al.^18^ pointed out that green TP EGCG and the 67-kDa laminin receptor (67LR) can reduce the TLR4 expression in macrophages. Byun et al.^17^ also found that EGCG can inhibit TLR4 signaling through 67LR in LPS-stimulated dendritic cells. Last year, Kumazoe et al.^25^ uncovered that EGCG can suppress TLR4 expression by up-regulating E3 ubiquitin-protein ligase RNF216 in macrophages. All these findings had been verified with our results in Figure 4, that TP recognized by 67LR then down-regulate TLR4 in melanoma to inhibit the cell functions.

In summary, our study showed that TLR4 protein expression level in melanoma cells was significantly higher than that in normal skin cells, TP could decrease TLR4 protein expression levels in normal and activated (LPS) melanomas, TLR4 could enhance the proliferating, migrating and invading ability of melanoma cells and 67LR blocking could abolish the suppressions of TP on melanomas. There are some points deserved to discuss. TLR4 protein expression level in melanoma was high expressed and it could be down-regulated by TP. Since TLR4 in TP+67LR-shRNA group was higher than TP group, other substances that might recognize TP remained to be discussed in future studies. This article only testified TP significantly suppressed TLR4 expression and most TP could be recognized by 67LR, but the mechanism of TLR4 signaling pathway were still uncovered, which was the limitation of this study as well as the focus of future study.

The relationship among TP, TLR4, and melanoma had never been discussed before, which was the novel point in this research. TLR4 inhibition could significantly suppress proliferation, migration, and invasion in melanoma. TP could inhibit TLR4 expression in vitro and in vivo experiments with dose and time dependence.
